# Supplementary material for: Prognostic Factors in Pseudomyxoma Peritonei with Emphasis on the Predictive Role of Peritoneal Cancer Index and Tumor Markers
Source: Cancers (Basel). 2023 Feb 19;15(4):1326. doi: 10.3390/cancers15041326 (PMC9954733; doi:10.3390/cancers15041326)
Supplement: Supplementary file 1 [file cancers-15-01326-s001.zip › cancers-2214608-supplementary.pdf]

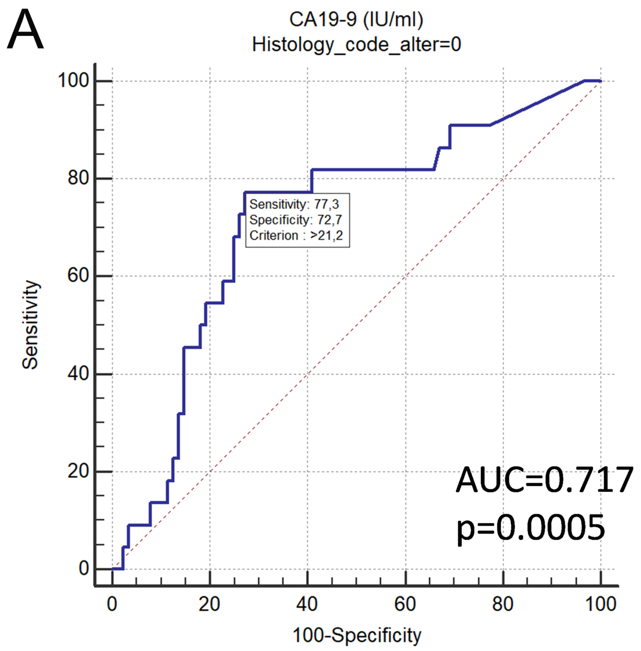

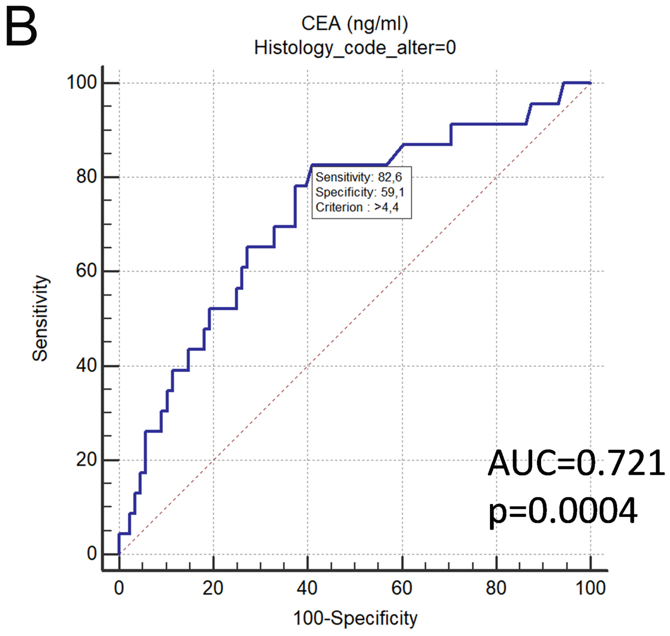

Supplementary Figure S1. ROC curve analysis for tumor markers. CA19-9 plasma level in LAMN patients predicts disease relapse with an AUC of 0.717 (p=0.0005, A) and with a sensitivity of 77.3% and specificity of 72.7%. CEA plasma level in LAMN patients predicts relapse with an AUC of 0.721 (p=0.0004, B) and with a sensitivity of 82.6% and specificity of 59.1%.

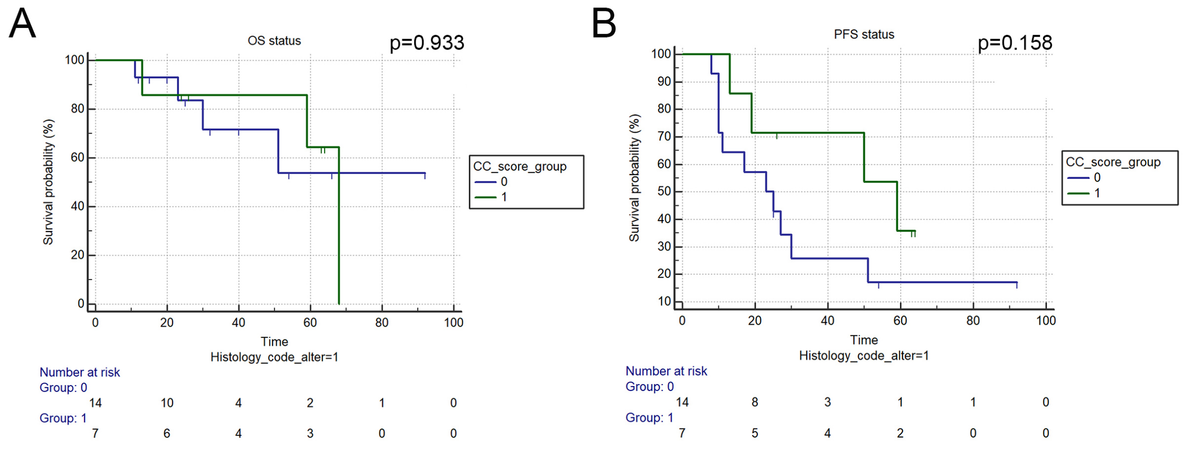

Supplementary Figure S2. Prognostic role of CC score in HAMN patients. CC score group (0-1 vs 2) was not prognostic neither for OS (p=0.933) nor for PFS in patients with HAMN (p=0.158).

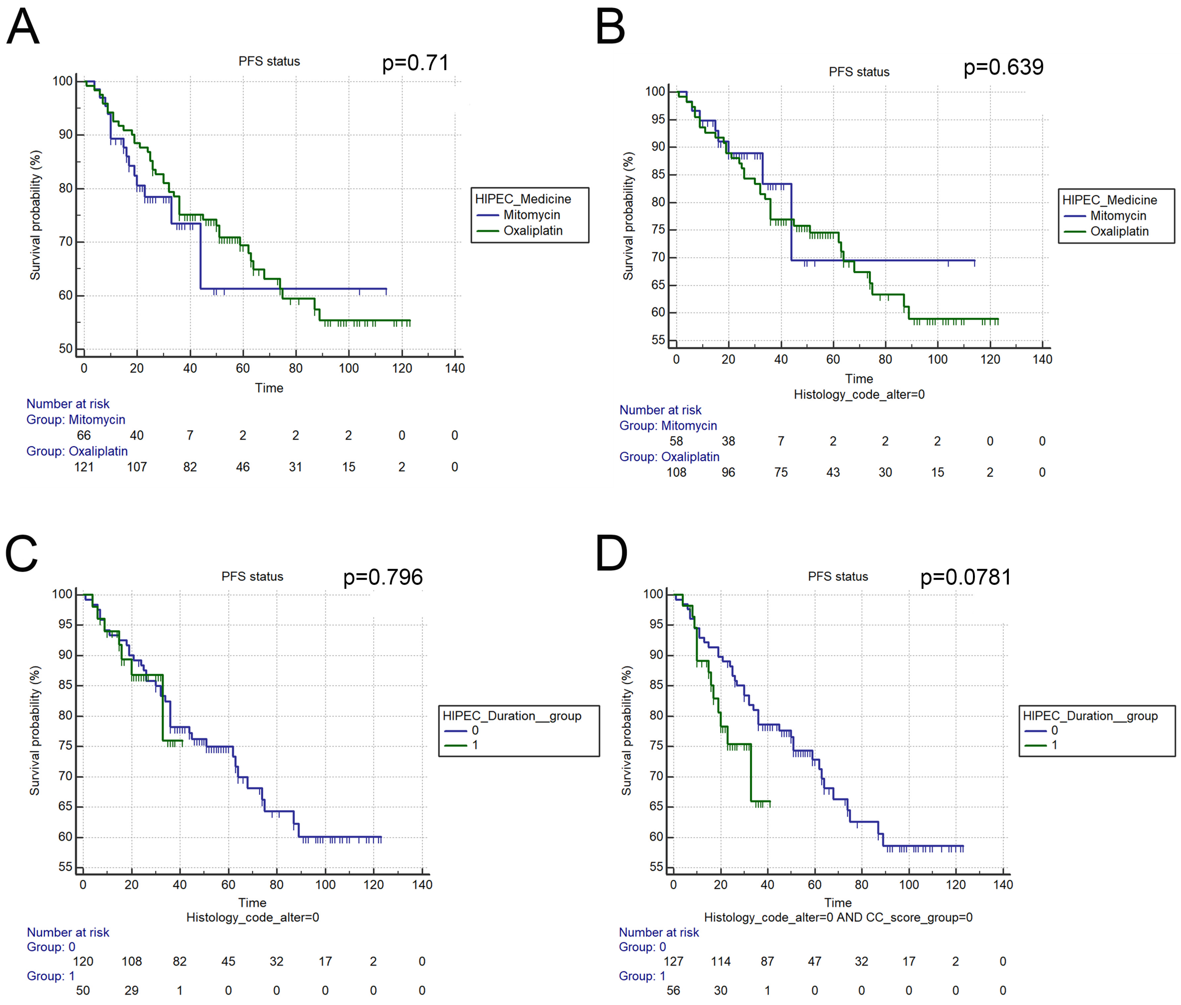

Supplementary Figure S3. Predictive role of HIPEC medicine and duration in PMP. Used HIPEC medicine (Mitomycin vs Oxaliplatin) was not predictive regarding PFS neither in the whole cohort

(p=0.71, A) nor in LAMN patients (p=0.639, B). There was no significant difference in the PFS of patients according to the duration of HIPEC (30-60 mins vs 90 mins) neither in the whole LAMN cohort (p=0.796, C), nor in LAMN patients with CC0-1 situation (p=0.0781, D).

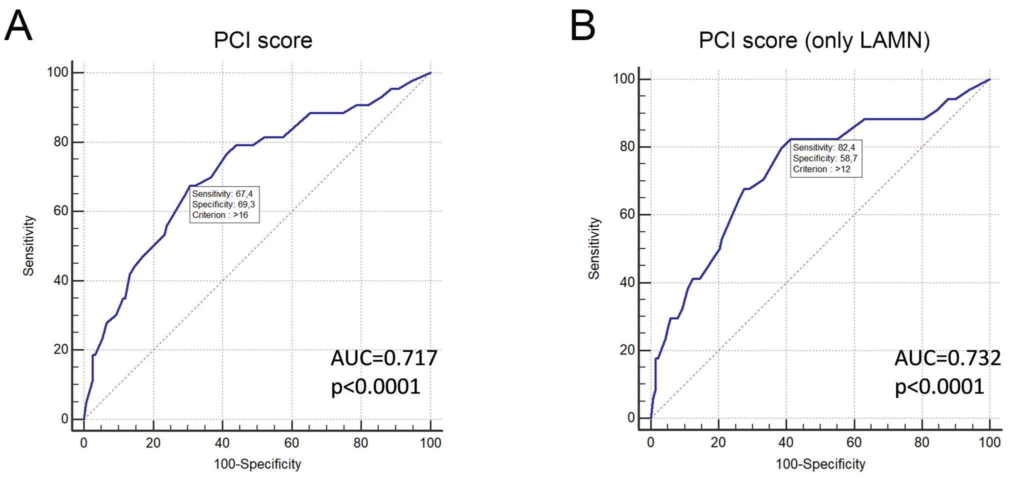

Supplementary Figure S4. ROC curve analysis for PCI. PCI in the whole cohort predicts disease relapse with an AUC of 0.717 (p<0.0001, A) and with a sensitivity of 67.4% and specificity of 69.3%. PCI in LAMN patients predicts relapse with an AUC of 0.732 (p<0.0001, B) and with a sensitivity of 82.4% and specificity of 58.7%.
